# Teleconsultation in children with abdominal pain: a comparison of physician triage recommendations and an established paediatric telephone triage protocol

**DOI:** 10.1186/1472-6947-13-110

**Published:** 2013-09-30

**Authors:** Gabrielle Marmier Staub, Jan von Overbeck, Eva Blozik

**Affiliations:** 1 Swiss Center for Telemedicine Medgate, Gellertstr. 19, 4020 Basel, Switzerland; 2 Department of Primary Medical Care, University Medical Center Hamburg-Eppendorf, Martinistr. 52, 20246 Hamburg, Germany

## Abstract

**Background:**

Quality assessment and continuous quality feedback to the staff is crucial for safety and efficiency of teleconsultation and triage. This study evaluates whether it is feasible to use an already existing telephone triage protocol to assess the appropriateness of point-of-care and time-to-treat recommendations after teleconsultations.

**Methods:**

Based on electronic patient records, we retrospectively compared the point-of-care and time-to-treat recommendations of the paediatric telephone triage protocol with the actual recommendations of trained physicians for children with abdominal pain, following a teleconsultation.

**Results:**

In 59 of 96 cases (61%) these recommendations were congruent with the paediatric telephone protocol. Discrepancies were either of organizational nature, due to factors such as local referral policies or gatekeeping insurance models, or of medical origin, such as milder than usual symptoms or clear diagnosis of a minor ailment.

**Conclusions:**

A paediatric telephone triage protocol may be applicable in healthcare systems other than the one in which it has been developed, if triage rules are adapted to match the organisational aspects of the local healthcare system.

## Background

Telephone consultations and triage have increasingly been used during the last few years as a means for healthcare delivery [1,2]. Almost half of patients can manage their health problem themselves after teleconsultation, and do not need a face-to-face consultation [3]. In addition to reducing unnecessary physician or emergency room visits, one motive for teleconsultation services is to help identify patients with serious medical problems and ensure they receive timely and adequate care, from the appropriate type of health care provider [4-7]. Health care professionals doing teleconsultation and triage are largely limited to medical history taking, without access to other diagnostic tools. Thorough and correct history taking is therefore of essential importance. Based on this, health professionals evaluate differential diagnoses, and judge the urgency of the most likely diagnosis and an acceptable level of uncertainty, taking into account the possible risks. In general, "it is easier to identify an emergency than to rule one out" [8]. Even well-trained physicians omit important questions necessary for triage unless prompted by some sort of decision support such as a written protocol or computerized algorithm [9]. Telephone triage protocols are intended to assist health professionals to structure history taking, therefore increasing the efficiency and safety of triage [3,4,10,11].

Quality assessment and continuous quality feedback to the staff is crucial for safety and efficiency of teleconsultation and triage [12]. Currently, quality assessment in our centre is done based on a complex, time-consuming evaluation of random call records [13]. For decreasing the required resources for quality assessment, we aimed at evaluating whether it is feasible to use an already existing and widely accepted telephone triage protocol to assess the appropriateness of actual point-of-care and time-to-treat recommendations after teleconsultations [14-16]. For this purpose, we used the concrete example of abdominal pain in children which is a frequent reason for encounter in primary paediatric care, both in face-to-face and in teleconsultation settings. It requires a thorough investigation by a health professional primarily because it may be associated with underdiagnosed serious conditions such as appendicitis [9,17]. Specifically, based on index symptoms for abdominal pain in children, we compared the point-of-care and time-to-treat recommendations of experienced physicians trained in telemedicine with the recommendations as listed in a broadly accepted and widely used telephone triage [15].

## Methods

### Setting

In 2011, the Swiss Center for Telemedicine (Medgate) provided up to 400 teleconsultations per day, mainly done over the phone. Consultations covered the whole spectrum of medical problems that are common in the general practice setting. Our services are freely available to all patients whose health insurance companies have a service agreement with Medgate, currently including well over half of the Swiss population. The present evaluation was approved by the local ethics committee (Ethics Committee of Basle, EKBB).

### Telemedical processes at the Swiss Centre for Telemedicine Medgate

When patients or proxies contacted the telemedicine centre for a medical consultation, a call centre agent (non-medical personnel) recorded personal details and transferred the call to a telemedical assistant (trained paramedical personnel). In case of severe symptoms or signs of a life-threatening emergency, the call was directly transferred to a medical doctor. In non-emergency cases the telemedical assistant assessed the urgency of the medical problem and scheduled an appointment for a call-back by a physician trained in telemedicine, who then conducted a detailed teleconsultation. A multidimensional quality assurance system is in place, including the training of new employees in clinical telemedicine, continuing education of medical and non-medical staff, supervision by experienced team members, and a standardized evaluation of teleconsultations and case reviews. Clinical checklists for the most frequent reasons for encounter in our setting were developed including lists of questions that should be asked, of differential diagnoses that need to be considered, of alarm symptoms, and medications that are appropriate to be prescribed telemedically.

### Patient population and source of data

Between calendar week 26 and 33 in 2011, all consecutive medical teleconsultations for children (<18 years of age) whose parents or other proxies reported abdominal pain to be the major symptom or complaint were selected and analysed retrospectively.

### Measures

The broadly accepted and widely used paediatric protocol for abdominal pain published by Schmitt and was used as a basis [15]. This protocol was selected because it has been issued by the American Academy of Pediatrics, a not-for-profit professional organization, it is intended to be used by all type of health professionals not only by telenurses, and it is the most widely known and broadly accepted paediatric telephone protocol in Europe.

In a first step, we extracted all index symptoms (for example "Signs of shock", "Pain on the low right side") listed in the paediatric telephone protocol for abdominal pain (Please see left column of Table 1 for details). Secondly, for each case included in the present analysis, a physician specialized in internal medicine and trained in quality assurance reviewed the case documentation in the individual electronic patient record and assigned the most appropriate index symptom from this list to the individual case. If the information in the electronic patient record was unclear the audio recording of the teleconsultation was analysed. To check, whether the process of retrospectively assigning index symptoms to a case is reliable, two physicians independently assigned index symptoms for a random sample of cases. This procedure did not show any interrater differences in classifying the patient’s symptoms. In a third step, the characteristics of the case (time of day, age of patient, gender) and the triage recommendations that have been actually given by the physician over the phone were extracted from the electronic patient records. Fourthly, the index symptoms that have been assigned to each case were associated with the corresponding triage disposition (i.e. "Call emergency services now", "Go to emergency department now", "Go to office now", "See today in office", "See within 2 weeks in office", "Home care") as recommended in the paediatric telephone triage protocol. Fifthly, for all cases with discrepancies between the triage disposition as listed in the paediatric telephone protocol and the actual triage recommendation of the Medgate physician, the potential reason for the discrepancy (organizational versus medical) was analysed by two reviewers independently. Discrepant results across reviewers were resolved by discussion. Finally, a paediatrician experienced in quality control reviewed the medical appropriateness of the recommendation in all cases that were discrepant for non-organizational reasons.

**Table 1:** **Characteristics of cases included in the present analysis** (**N**=**96**)

**Characteristic**	**N**	**%**
Out of hours	50	52%
Age of patient (mean ± standard deviation)	5.8 ± 3.7 years	
(range)	3 weeks – 15 years	
Female gender	51	53%
**Clinical triage criteria (Barton Schmitt)**		
***Call emergency services now***		
Signs of shock (e.g. very weak, limp, not moving, gray skin)	0	0%
Sounds like life-threatening emergency to the triager	1	1%
***Go to emergency department now***		
Vomiting blood (R/O peptic ulcer, esophagitis)	0	0%
Is pregnant or could be pregnant (female) (R/O spontaneous abortion, ectopic pregnancy)	0	0%
Could be poisoning with a plant, medicine, or chemical	0	0%
Lying down and unable to walk (R/O appendicitis or other acute abdomen)	1	1%
Walks bent over or holding the abdomen (R/O appendicitis or other acute abdomen)	7	7%
Pain in the scrotum or testicle (male) (R/O testicular torsion)	0	0%
Severe (excruciating) pain (R/O serious cause)	10	10%
Blood in the bowel movements (R/O peptic ulcer, intussusception)	0	0%
Sounds like intussusception to the triager (attacks of severe abdominal pain/crying suddenly switching to 2 to 10 minute periods of quiet) (age usually < 3 years)	1	1%
Child sounds very sick or weak to the triager (R/O serious cause)	0	0%
***Go to office now***		
Pain low on the right side (R/O appendicitis)	6	6%
Pain (or crying) that is constant for > 2 hours (R/O appendicitis, PID, other serious cause)	21	22%
Vomiting bile (bright yellow or green) (R/0 intestinal obstruction)	1	1%
Recent injury to the abdomen (R/O ruptured spleen, traumatic pancreatitis)	0	0%
Tenderness mainly present low on right side when caller presses on the abdomen	5	5%
Age < 2 years (R/O intussusception, especially with intermittent pain)	7	7%
Fever > 40.6°C (R/O serious bacterial infection)	1	1%
***See today in office***		
Mild pain that comes and goes (cramps) lasts > 24 h	20	21%
Parent wants child seen	1	1%
***See within 2 weeks in office***		
Abdominal pains are a chronic problem (present > 4 weeks) (R/O stress-related, school avoidance, normal sideache)	4	4%
***Home care***		
Mild abdominal pain present for < 24 h	10	10%

### Data analysis

Data analysis was completely descriptive and included simple counting and frequency distributions of the demographic and clinical characteristics of the participants and of outcome measures. Data were analysed using Microsoft Excel 2010 (http://www.microsoft.com).

## Results

### Characteristics of the study sample

A total of 96 patients were included in the analysis. About half of teleconsultations were out of hours. The mean age of the patients was 5.8 ± 3.7 years, and age ranged between 3 weeks and 15 years. Index symptoms as based on Schmitt’s paediatric telephone protocol are listed on Table 1. The most prevalent symptoms were pain or crying that was constant for > 2 hours (22% of children), mild pain that came and went (cramps) lasting > 24 h (21%), severe pain (10%), and mild abdominal pain present for < 24 h (10%).

### Accordance of triage recommendations after medical teleconsultation and paediatric telephone triage protocol

Table 2 depicts the actual triage recommendations of the physicians after teleconsultation in comparison with the recommendations of the paediatric telephone triage protocol. In 59 cases (61%) the triage recommendations of physicians were congruent with those of the protocol.

**Table 2:** **Accordance of physician triage recommendations in comparison to a paediatric telephone triage protocol for abdominal pain** (**N**=**96**)

	**Physician triage recommendation**					
**Triage according to paediatric telephone protocol for abdominal pain**	Emergency services called	Go to emergency department now	Go to office now	See today in office	See within 2 weeks in office	Home care
Call emergency services now, N	1					
Go to emergency department now, N	1	14	4			1
Go to office now, N		11	21	3	1	4
See today in office, N		3	2	12	1	3
See within 2 weeks in office, N				2	2	
Home care, N				1		9
Total, N	2	28	27	18	4	17
Physician triage recommendation in accordance with telephone triage protocol, N (%)	1 (50%)	14 (50%)	21 (78%)	12 (67%)	2 (50%)	9 (53%)
Logistic/organisational reasons for discrepancy, N (%)	1 (50%)	11 (39%)	4 (15%)	0 (0%)	0 (0%)	0 (0%)
Medical reasons for discrepancy, N (%)	0 (0%)	3 (11%)	2 (7%)	5 (28%)	2 (50%)	8 (47%)
Inappropriate management by physician, N (%)	0 (0%)	0 (0%)	0 (0%)	1 (5%) (overtriage)	0 (0%)	0 (0%)

### More conservative recommendations

In 20 cases (21%) of patients, the physicians recommended more conservative measures than the protocol. For the most part (N=12), this was due to organizational rather than medical reasons:

•1 patient was in a mountain shelter away from the road, so the parents were not able to get to a hospital without the help of emergency rescue services. The physician therefore recommended calling emergency services, whereas the protocol recommended "Go to emergency department now".

•In 6 patients, the physician on the phone suspected the presence of appendicitis or another serious cause for an acute abdomen. Based on the paediatric telephone triage protocol these patients would be sent to a doctor’s office. However, the usual procedure in Switzerland is to directly transfer such patients to hospital care.

•1 patient who would have been sent to an office according to the triage protocol was dehydrated in addition to the presence of abdominal pain. In Switzerland, dehydrated patients are usually managed in hospital care.

•Four teleconsultations were out of hours and the paediatrician in private practice was not available. In these situations, patients are usually transferred to the next emergency department.

In 8 additional cases, the physician on the phone triaged more conservatively because he was insufficiently sure of the diagnosis, e.g. due to proxy’s inability to communicate in the national languages or in English. None of these discrepant cases were managed inadequately according to the paediatrician’s case review.

### Medical problem considered less urgent

Based on the teleconsultation, in 17 out of 96 cases (18%) physicians considered the problem to be less urgent than the recommendations of the protocol. There were medical reasons in 8 of these 17 cases. Specifically, Medgate physicians recommended homecare as opposed to a face-to face encounter in the following situations:

•1 patient with significant improvement of abdominal pain after defecation

•1 baby with colic

•1 baby after replacement of breastfeeding by cereal meals

•3 patients with gastroenteritis

•1 patient with mild symptoms

•therapy on probation after explanation of alarm symptoms, and for an a priori defined period of time, in 1 patient with suspicion of functional abdominal pain

Additionally, 1 patient with gastroenteritis,1 patient with mild symptoms and 1 patient with suspected intolerance of food were recommended to be seen in the office today, as opposed to the protocol’s recommendation of going to the office immediately.

Five additional children were transferred to a family physician instead of hospital care because they had a special gatekeeper health insurance model. These persons had to consult a fixed general practitioner in all situations except life-threatening emergencies and preventive services.

One case was managed suboptimally because the physician on the phone did not clearly enough communicate his time-to-treat recommendation. As a result, the patient went to the office later the same day instead of going immediately. All other cases were managed appropriately, according to the paediatrician’s clinical review.

## Discussion

The present study indicates that physician triage recommendations are congruent to a widely accepted telephone triage protocol for the majority (61%) of teleconsultations in children with abdominal pain. However, there also seem to be considerable systematic differences between "real-life" triage recommendations and protocol triage criteria. These differences may be categorized as being either of organizational (N=16, 44% of discrepant cases) or of medical origin (N=20, 56% of discrepant cases).

One aspect that needs to be considered when interpreting the results of the present study is that the organization of the Swiss healthcare system differs from the system in the U.S.A. for which the paediatric telephone triage protocol was initially developed [18]. For example, in Switzerland patients with a high probability of having appendicitis are usually transferred directly to hospital care because of the availability of a surgery department, whereas the U.S.A. protocol has them referred to a doctor’s office. Additionally, a significant number of persons in Switzerland choose alternative health insurance models. In return for a reduction in premiums, these persons abandon the free choice of healthcare providers, and have their access to care controlled by a gatekeeper, typically a GP or a teleconsultation centre. This had implications for the subgroup of patients included in this study which had access to our services, but had their GP as their gatekeeper. These persons had to be referred to their GP, even though referral to another healthcare provider would have been appropriate from the medical perspective. These differences in health system factors limit the generalizability of the present results. In addition, there may be cultural factors, such as differences in attitudes towards use of health services which differ across countries and may influence triage recommendations. Using triage protocols for quality assessment in other healthcare systems should thus be carefully piloted.

Moreover, the telephone triage protocols used as a basis for the present analysis were designed for use by a wide range of medical personnel, including nurses, nurse practitioners and physician assistants in addition to physicians. Given the fact that physicians have much broader possibilities to do teleprescription or telediagnostics than less highly qualified personnel, one might hypothesize that there are more clinical situations in which it is appropriate for physicians to recommend selfcare [3]. This may explain why almost half of the cases in which physicians in our setting recommended selfcare would have been referred to face-to-face care according to the triage protocol (none of these selfcare recommendations were rated inappropriate by the reviewing paediatrician). The type and qualification of healthcare personnel performing teleconsultation and triage services vary across countries. In Denmark, for instance, such services are provided by general practitioners, [19] while in Great Britain and Sweden these services are conducted by nurses who are strictly guided by protocols [20]. These differences in staff need to be considered when discrepancies between the protocol and the actual triage decisions are interpreted.

There are several limitations that have to be considered when evaluating this research. We focused on abdominal pain and can therefore draw no conclusions regarding other symptoms. The analysis was done on children, so the results may not be generalizable to adult patient populations. For budgetary reasons, we analysed a comparatively small number of cases, which may decrease the precision of our results. Large-scale studies in different healthcare settings are needed before results of this study should be generalised to other health systems. However, we analysed all consecutive patients receiving a medical teleconsultation for abdominal pain in a defined time interval, giving good coverage of the spectrum of patients and symptoms. Information for data analysis was extracted retrospectively from the electronic patient record and the recorded teleconsultation. This may have led to imprecision, but not to bias. However, the strength of our study is that data come from routine services running with trained and experienced personnel, under a sophisticated quality assurance system, and can therefore be judged as reliable. Finally, the present study was done in one specific centre in Switzerland, and the population of callers may be subject to selection bias. More than half of the Swiss population from all parts of the country have access to our services, so we assume that the paediatric patient population at our centre is largely representative for the paediatric primary care setting in Switzerland. Overall, it has to be emphasised that this is an explorative analysis using descriptive statistics only. Future studies should be designed for hypothesis testing (inferential statistics).

Quality standards and quality management are crucial for safety and effectiveness of teleconsultation and triage [12]. For example, Derkx and colleagues evaluated quality of calls at Dutch out of hours centres using telephone standardised patients. They found that the appropriate triage outcome was achieved only in 58% of calls [21]. The present study design assumes that experienced physicians trained in telemedicine who work under a comprehensive quality assurance framework and whose discrepant recommendations were assessed to be appropriate by a paediatric quality reviewer are the gold standard for teleconsultation and triage in our setting [22]. The difficulty of evaluating the appropriateness of triage recommendations is that there is no commonly accepted gold standard for assessing quality in this setting. For example, van Ierland and colleagues evaluated telephone in comparison to face-to-face emergency triage and concluded that a reference standard for comparing the appropriateness of triage recommendations is missing [23]. The aim of the present study was to evaluate whether it is feasible to use an already existing telephone triage protocol to assess the appropriateness of point-of-care and time-to-treat recommendations after teleconsultations. For this purpose, it is inherent that the protocol is used retrospectively as are quality assessments usually done. This means that we used the paediatric protocol in a different way as it was initially developed for, and we cannot conclude on the feasibility and validity of using the protocol as a guide for triage. This would need another study design such as a before/after or a head-to-head implementation study.

This study focused on the appropriateness of triage recommendations without evaluating adherence to recommendations or outcome in terms of health or costs. Previous research has shown that compliance with telephone triage recommendations is generally high [24,25], and several studies including randomized controlled trials and systematic reviews have indicated that teleconsultation and triage are safe and effective [1-6,22]. An assessment of the appropriateness of medial triage recommendations, irrespective of the outcome, may be helpful to appraise the quality of institutions or individual healthcare providers.

## Conclusions

Telephone triage protocols were not designed for quality assessment of triage and were meant to be used by trained and experienced medical personnel (usually nurses) in the context of a live call. Using a protocol retrospectively on a document or audiotape without the nurse and patient able to have explanatory and supplemental dialog is taking the protocol out of its intended clinical context. However, the present study evaluates whether already existing protocols may be used as an independent reference standard to evaluate the appropriateness of triage recommendations in a pilot design. Based on our findings we conclude that it is feasible to use triage criteria from established protocols given triage criteria are adapted to account for the characteristics of the local healthcare system.

## Competing interests

GMS, JVO, and EB are employees of the Swiss Center for Telemedicine Medgate. The study was funded by the Swiss Center for Telemedicine, Basel, Switzerland. The sponsor had no role in design, in the collection, analysis, and interpretation of data; in the writing of the manuscript; and in the decision to submit the manuscript for publication.

## Authors’ contributions

GMS participated in the design of the study, collected data, and drafted the first version of the manuscript. JVO participated in data interpretation and critically revised the manuscript. EB conceived of the study, and participated in its design and coordination and helped to draft the manuscript. All authors read and approved the final manuscript.

## Pre-publication history

The pre-publication history for this paper can be accessed here:

http://www.biomedcentral.com/1472-6947/13/110/prepub
